# SLJ is associated with multiple field-based fitness indicators in young adult males

**DOI:** 10.3389/fpsyg.2026.1856443

**Published:** 2026-07-09

**Authors:** Chao-Fu Chen, Hui-Ju Wu, Yan-Zhi He, Jian-Fu Cheng, Bo-Jun Zhou, Jian-Ming Wu, Qi-Fan Zheng, Xin-Rui Wang, Teng-Xiang Ge, Lin-Zhu, Jian-Hua Pan

**Affiliations:** 1 Physical Education College, Jimei University, Xiamen, China; 2 Xiamen Yixue Sports and Technology Co., Ltd., Xiamen, China; 3 Physical Education Department, Xiangtan University, Xiangtan, China; 4 Physical Education College, Zhengzhou University of Industrial Technology, Zhengzhou, China; 5 College of Physical Education, Chongqing University, Chongqing, China

**Keywords:** cardiometabolic health, college students, physical fitness, sports, standing long jump

## Abstract

**Introduction:**

Health data equity emphasizes the need for accessible, low-cost, and scalable indicators that can reduce barriers to health assessment when applied within clearly defined populations and contexts. However, many existing fitness and health evaluation methods rely on resource-intensive measurements, limiting their applicability in large-scale or under-resourced settings. The standing long jump (SLJ), a simple field-based test of lower-limb explosive power, may serve as a practical and scalable indicator of physical fitness among male college students.

**Methods:**

This cross-sectional study included 7,842 male college students. SLJ performance was assessed alongside key health-related fitness indicators, including cardiorespiratory fitness (1000-m run), muscular endurance (pull-ups), flexibility (sit-and-reach), and body composition (BMI). Pearson correlation analysis was conducted to examine associations between SLJ and these variables.

**Results:**

SLJ performance was significantly associated with all measured variables (*p* < 0.01). It was negatively correlated with 1,000-m run time and BMI, suggesting associations with better cardiorespiratory fitness performance and lower BMI within this male college student sample. Positive correlations were observed between SLJ and both pull-up performance and flexibility. These findings indicate that SLJ is associated with multiple dimensions of physical fitness in young adult men.

**Conclusion:**

As a low-cost, scalable, and easily implementable measure, SLJ has potential utility for large-scale fitness assessment among male college students. Its associations with multiple domains of physical fitness support its use as an accessible field-based assessment tool in university or resource-limited settings. Given that the present study included only male college students, the findings should not be generalized to female students, other age groups, or non-college populations without further validation. These findings contribute to the development of accessible and data-efficient strategies for context-specific health and fitness assessment.

## 1 Introduction

Lifestyle-related behaviors, including physical activity, exercise participation, and sedentary behavior, are widely recognized as important factors associated with cardiometabolic and mental health (Warburton and Bredin, 2017; Guthold et al., 2018). Regular physical activity and higher levels of physical fitness are associated with lower risks of obesity, cardiovascular disease, and metabolic disorders, as well as better mental health outcomes, including lower levels of depression and anxiety (Pedersen and Saltin, 2015; Biddle et al., 2019). Conversely, insufficient physical activity and prolonged sedentary behavior are closely linked to poorer physical fitness and adverse health-related outcomes (Owen et al., 2010; Hallgren et al., 2020). Therefore, practical and scalable methods for assessing health-related physical fitness are important for school-based, university-based, and large-scale fitness monitoring.

Health-related physical fitness is commonly understood as a multidimensional construct that includes cardiorespiratory fitness, muscular strength and endurance, flexibility, and weight-related indicators (Ortega et al., 2008; Blair et al., 2001). These components are frequently used to evaluate physical fitness status and to support health promotion and exercise guidance at both individual and group levels (Anderson and Durstine, 2019; Ruiz et al., 2009). Among these components, cardiorespiratory fitness and weight status have been widely examined in relation to cardiometabolic and mental health outcomes (Ross et al., 2016; Ortega et al., 2015; Kandola et al., 2019). However, comprehensive assessment of multiple fitness components often requires several tests, trained personnel, sufficient space, and standardized procedures, which can increase the time and resource burden of large-scale fitness evaluation (Colley et al., 2019).

In large-scale populations, such as school or university fitness surveillance systems, comprehensive physical fitness testing may be difficult to implement efficiently. This highlights the need for simple, low-cost, and field-based tests that can be administered easily and repeatedly in practical settings (Arboix-Alió et al., 2024; Lang et al., 2018). The standing long jump (SLJ), a commonly used measure of lower-limb explosive performance, is characterized by its simplicity, minimal equipment requirements, and ease of implementation, making it suitable for large-scale and resource-limited assessment contexts (Castro-Piñero et al., 2010; Markovic et al., 2004). Previous studies have shown that lower-limb explosive performance is associated with several physical fitness components, including muscular strength, speed, and athletic performance (Haff and Nimphius, 2012; Suchomel et al., 2016). However, evidence remains limited regarding whether SLJ performance is concurrently associated with multiple health-related physical fitness indicators in large samples of male college students (Piqueras et al., 2021; Mohajan and Mohajan, 2023).

Therefore, this study aimed to examine the associations between SLJ performance and multiple health-related physical fitness components, including cardiorespiratory fitness, muscular endurance, flexibility, and BMI-defined weight status, in a large sample of male college students. We hypothesized that SLJ performance would be significantly associated with these fitness indicators. Given its low cost, simplicity, and scalability, SLJ may provide supplementary information within a broader field-based physical fitness assessment system, rather than serving as a standalone measure of overall health status or health disparities.

## 2 Materials and methods

### 2.1 Participants

This study employed a cross-sectional design and included male undergraduate students from first to fourth year, aiming to provide a large-scale and population-relevant dataset for health-related fitness analysis. A total of 9,314 participants were initially recruited, reflecting a broad sample suitable for population-level health assessment. To ensure data quality and the stability of the results, individuals with missing data or those unable to complete the required tests were excluded.

Additionally, participants with abnormal body mass index (BMI) values (BMI < 18.5 or BMI > 28) were excluded to obtain a relatively homogeneous and healthy sample for analysis. A final sample of 7,842 participants was included. Among them, 1,601 were first-year students, 1,914 were second-year students, 2,104 were third-year students, and 2,223 were fourth-year students.

The mean height, body mass, and BMI of the participants were 175.5 ± 5.6 cm, 69.2 ± 10.8 kg, and 22.4 ± 2.6 kg/m^2^, respectively. All participants were considered healthy, with no known cardiovascular diseases, metabolic disorders, or musculoskeletal injuries.

The large sample size and standardized data collection procedures enhance the reliability and scalability of the dataset, supporting its potential application in population-level health monitoring and contributing to more accessible and equitable health data generation.

### 2.2 Experimental procedure

All assessments were conducted on a standard athletics field using field-based testing protocols. This study aimed to examine the relationships between standing long jump (SLJ) performance and multiple lifestyle-related physical fitness components, including cardiorespiratory fitness, muscular endurance, flexibility, and body composition.

As a simple and field-based indicator of lower-limb explosive power, the SLJ reflects neuromuscular function and whole-body coordination, and represents an important component of lifestyle-related physical fitness. Importantly, its minimal equipment requirements and ease of administration make it highly suitable for large-scale implementation and accessible health data collection across diverse populations.

The selected fitness components—cardiorespiratory fitness, muscular endurance, flexibility, and body composition—are well-established health-related indicators closely associated with cardiometabolic and mental health. Together, these measures provide a multidimensional representation of physical fitness and overall health status.

All measurements were administered by trained physical education instructors following standardized protocols to ensure consistency, reliability, and reproducibility of the data. The use of standardized field-based assessments further supports the scalability of data collection and reduces barriers to implementation in resource-limited settings.

**Figure 1:**
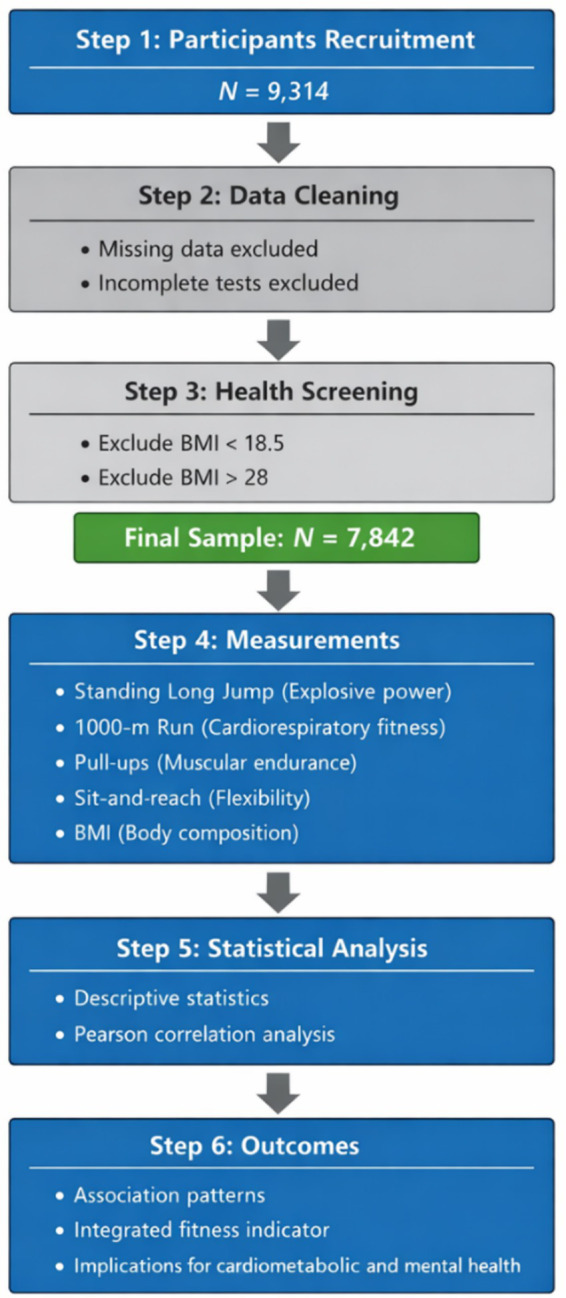
Technical flowchart of the study design and data analysis.

The overall technical framework of the study is illustrated in (Figure 1), highlighting the integration of field-based measurements into a structured and scalable health data acquisition pipeline.

### 2.3 Definition of outcome measures

The primary outcome measures were selected to provide a multidimensional and scalable assessment of health-related physical fitness using standardized field-based protocols. These tests were chosen because they are simple, low-cost, feasible for large-scale implementation, and commonly used in school and university-based physical fitness assessment systems. However, these measures should be interpreted as field-based indicators rather than laboratory-based or gold-standard assessments:

1. *Standing long jump, SLJ*: The standing long jump (SLJ) was used to assess lower-limb The standing long jump (SLJ) was used to assess lower-limb explosive performance, which is closely associated with neuromuscular function, lower-limb power, and motor coordination (Jochem et al., 2019; Chen et al., 2025; Chen and Wu, 2022). The test was performed using a standardized SLJ measurement device equipped with an infrared non-contact sensing system, which automatically recorded jump distance to improve measurement accuracy and minimize human error. Participants stood with both feet parallel behind the take-off line and performed a forward jump using coordinated arm swing and lower-limb force generation. Jump distance was measured from the take-off line to the nearest landing point. Each participant completed two trials, and the best performance was recorded for analysis. Standardized instructions were provided to ensure proper technique and to avoid invalid attempts, such as using a running start or stepping over the take-off line (Zhou et al., 2020). Given its simplicity, low cost, and minimal equipment requirements, SLJ is suitable for large-scale field-based physical fitness assessment.
2. *Cardiorespiratory fitness*: Cardiorespiratory fitness performance was assessed using a 1,000-m running test. This test is a practical field-based measure commonly used in large-scale student fitness assessment systems to reflect endurance-running performance and aerobic-related fitness (Garcia-Hermoso et al., 2023; Mayorga-Vega et al., 2016). However, it should not be considered a gold-standard measure of cardiorespiratory fitness. Direct laboratory-based measurement of maximal oxygen uptake (VO₂max) provides a more precise assessment of cardiorespiratory fitness but is less feasible in large-scale testing due to requirements for specialized equipment, trained personnel, and greater testing time. The 1,000-m running test was conducted on a standard athletics track, and participants were instructed to complete the distance at maximal effort. Trained personnel supervised the test and recorded completion time in minutes and seconds. To improve data consistency and comparability, all participants were tested under similar environmental conditions and completed a standardized warm-up protocol. Therefore, in the present study, 1,000-m running time was interpreted as a field-based indicator of endurance-running performance rather than a direct measure of VO₂max.
3. *Muscular endurance*: Muscular endurance performance was assessed using a pull-up test, which reflects the endurance capacity of the upper limbs and trunk musculature (Marques et al., 2021; Fraser et al., 2021). Participants grasped a horizontal bar using an overhand grip and performed repetitions from a fully extended arm position to a chin-over-bar position, followed by a controlled descent. To ensure standardization and data reliability, participants were required to perform continuous and controlled movements without using momentum, such as swinging. Only correctly executed repetitions were counted as valid (García-Hermoso et al., 2021). It should be noted that pull-up performance is influenced not only by upper-body muscular endurance but also by body mass, because participants must lift their own body weight during each repetition. Therefore, individuals with higher BMI-defined weight status may be disadvantaged in this test, which may partially confound the association between pull-up performance and BMI. Accordingly, pull-up results were interpreted as field-based muscular endurance performance rather than an isolated measure of muscular capacity independent of body size.
4. *Flexibility*: Flexibility was assessed using the sit-and-reach test, a widely used field-based method for evaluating the flexibility of the posterior lower-limb muscles and lower back (García-Hermoso et al., 2021; Mayorga-Vega et al., 2014). The test was conducted using a standardized sit-and-reach device (TSN100-TQ), which automatically recorded maximal reach distance in centimeters. Participants sat with fully extended legs and feet placed against the testing board, then reached forward with both hands in a controlled manner. The best performance was recorded for analysis. This test has demonstrated good reliability and feasibility in large-scale population assessments (García-Hermoso et al., 2022). Nevertheless, the sit-and-reach test mainly reflects hamstring and lower-back flexibility and should not be interpreted as a comprehensive measure of whole-body flexibility.
5. *Body composition*: Weight status was assessed using body mass index (BMI), a simple and widely used anthropometric indicator calculated from height and body mass (García-Hermoso et al., 2022; Khanna et al., 2022). Height and body mass were measured using a standardized height–weight measurement device, which automatically calculated BMI in kg/m^2^. All measurements were conducted with participants wearing light clothing and no shoes to minimize measurement error. Although BMI is widely applied in large-scale health surveillance and population-based assessment because of its simplicity and low cost (Wu et al., 2024), it cannot distinguish fat mass from lean mass. Therefore, BMI was interpreted in this study as an indicator of weight status rather than a direct measure of body composition or cardiometabolic risk.

Overall, the selected outcome measures combine simplicity, standardization, and scalability, enabling efficient data collection in large samples. Although these field-based tests have known limitations compared with laboratory-based or more precise assessments, they remain acceptable for large-scale physical fitness evaluation when the primary objective is practical implementation rather than clinical diagnosis or direct physiological measurement.

### 2.4 Statistical analysis

Descriptive statistics were expressed as mean ± standard deviation (SD). Pearson correlation analysis was performed to examine the relationships between standing long jump (SLJ) performance and the various physical fitness indicators. The level of statistical significance was set at *α* = 0.05.

## 3 Results

**Figure 2:**
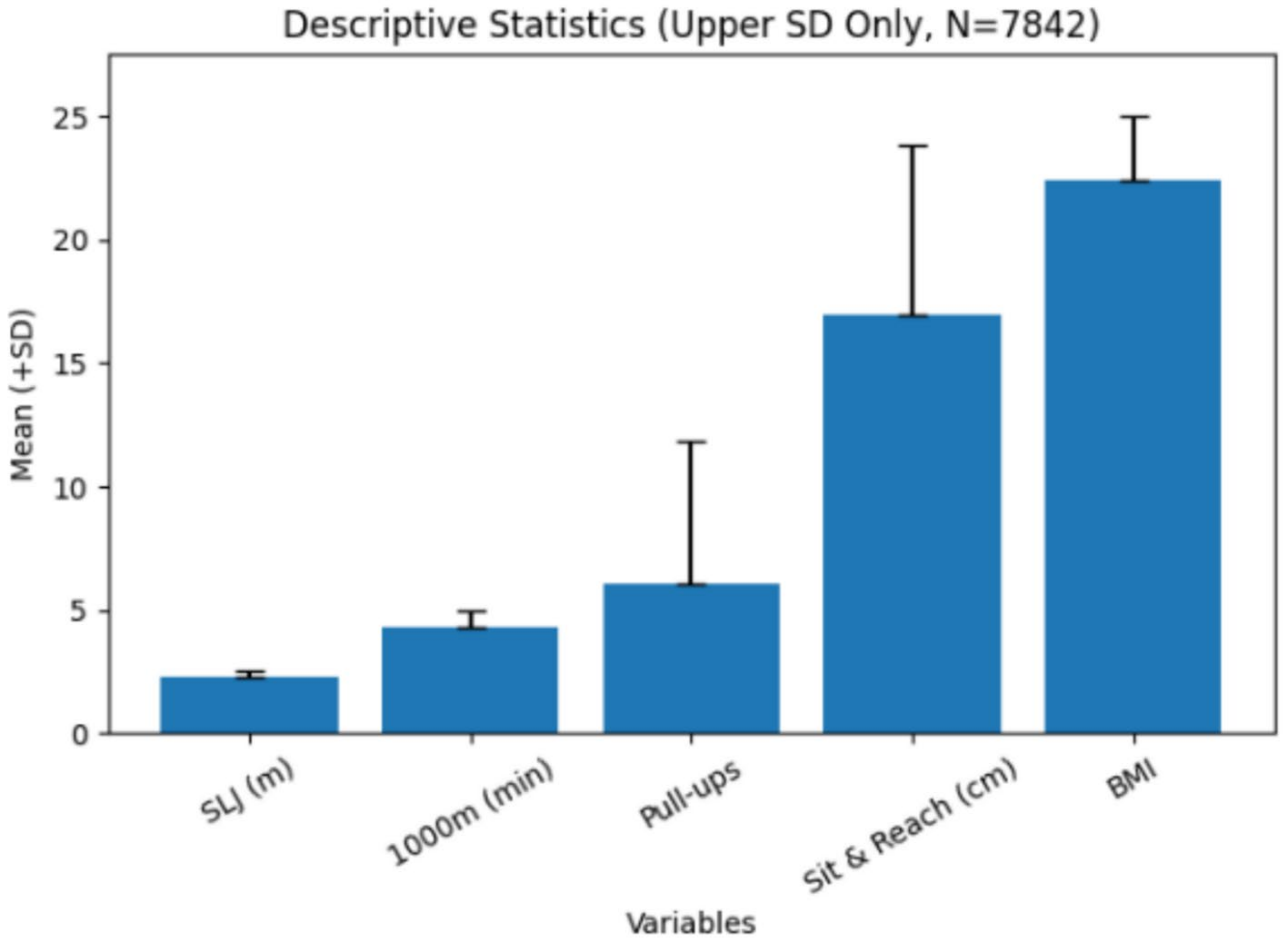
Standing long jump and health-related physical fitness indicators.

The descriptive statistics of standing long jump (SLJ) and all health-related physical fitness indicators are presented in Figure 2. The mean SLJ performance was 2.29 ± 0.24 m, while the average 1,000-m running time was 4.34 ± 0.60 min. The mean number of pull-ups was 6.05 ± 5.83 repetitions, and the average sit-and-reach score was 16.99 ± 6.85 cm. The mean body mass index (BMI) was 22.4 ± 2.60 kg/m^2^.

The correlation analysis showed that SLJ performance was moderately positively correlated with pull-up performance (*r* = 0.516, *p* < 0.01), explaining approximately 26.6% of the shared variance. This represents the strongest association observed in the present study and suggests that lower-limb explosive performance is meaningfully related to upper-body muscular endurance among male college students.

In contrast, the associations between SLJ and the other fitness-related indicators were statistically significant but small in magnitude. SLJ was positively correlated with sit-and-reach performance (*r* = 0.266, *p* < 0.01), explaining approximately 7.1% of the shared variance. SLJ was also negatively correlated with 1,000-m running time (*r* = −0.283, *p* < 0.01) and BMI-defined weight status (*r* = −0.284, *p* < 0.01), explaining approximately 8.0 and 8.1% of the shared variance, respectively. These findings indicate that students with better SLJ performance tended to have shorter 1,000-m running times, greater flexibility, and lower BMI values; however, the small effect sizes suggest limited predictive utility at the individual level.

**Figure 3:**
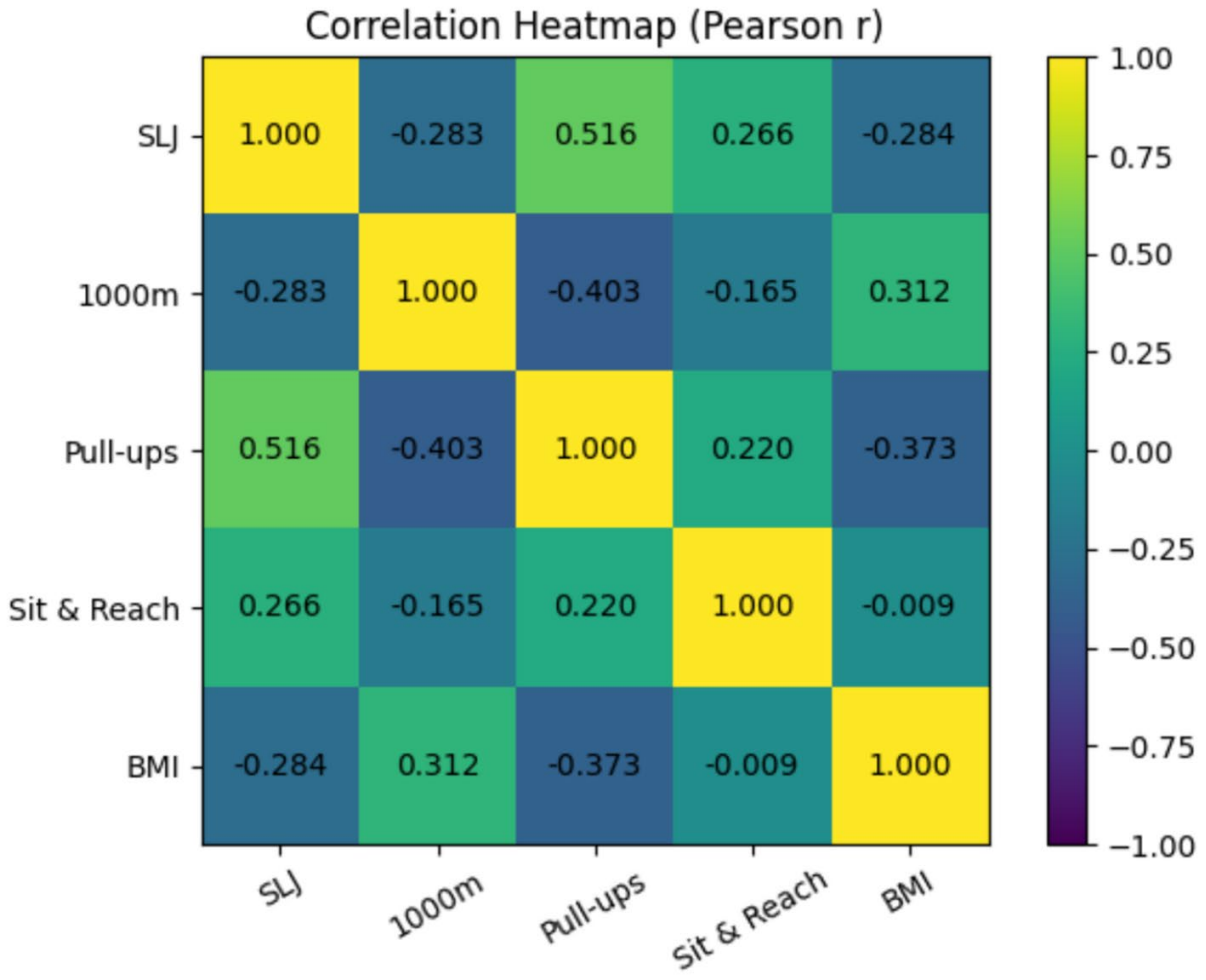
Correlation heatmap of standing long jump and physical fitness indicators.

Additionally, pull-up performance was moderately negatively correlated with BMI (*r* = −0.373), while 1,000-m running time was positively correlated with BMI (*r* = 0.312), suggesting that higher BMI-defined weight status may be associated with lower muscular endurance and slower endurance-running performance. Overall, although several correlations reached statistical significance, likely due in part to the large sample size, the relatively small shared variance for 1,000-m running time, BMI, and sit-and-reach indicates that SLJ should not be used as a standalone screening tool for these fitness components. Rather, SLJ may be more appropriately considered a simple field-based test that provides limited but useful supplementary information within a broader physical fitness assessment battery. These relationships are illustrated in (Figure 3).

## 4 Discussion

Based on a large sample of male college students, this study examined the associations between standing long jump (SLJ) performance and multiple components of health-related physical fitness. The results showed that SLJ performance was significantly associated with 1,000-m running time, pull-up performance, sit-and-reach performance, and BMI-defined weight status. Given the cross-sectional design of this study, these findings should be interpreted as concurrent associations rather than evidence of prediction, causation, or directionality. Within this context, SLJ may be considered a simple and scalable field-based measure that is related to several dimensions of health-related physical fitness among male college students, particularly in settings where comprehensive testing is difficult to implement.

These findings are consistent with previous research suggesting that health-related physical fitness is multidimensional and commonly includes cardiorespiratory endurance, muscular strength and endurance, flexibility, and weight-related indicators (McAvoy et al., 2026; Cancela-Carral et al., 2024). In the present study, SLJ was not interpreted as a direct measure of overall health status. Instead (Chiang et al., 2022; Leso et al., 2024), the observed associations suggest that SLJ performance is related to several physical fitness components assessed in this sample. Because no clinical biomarkers, disease outcomes, behavioral variables, or mental health measures were included, the findings should not be extended to cardiometabolic health, mental health, or overall health status. Rather, the practical value of SLJ lies in its simplicity, low cost, and feasibility as a field-based fitness test.

From the perspective of fitness structure and movement mechanisms, SLJ showed a moderate positive association with pull-up performance, representing the strongest association among the measured variables. This result suggests that lower-limb explosive performance and upper-body muscular endurance may coexist as related components of physical fitness in male college students. However, this association does not imply that improvement in one component causes improvement in the other. It may instead reflect shared contributions from general fitness level, neuromuscular function, training habits, or other unmeasured factors (Zhou et al., 2024). Additionally, the significant positive association between SLJ and sit-and-reach performance suggests that flexibility may be related to explosive movement performance, possibly through joint range of motion and movement efficiency. Nevertheless, because this study was correlational, the role of flexibility in SLJ performance should be interpreted cautiously (Bouamra et al., 2022).

SLJ performance was also significantly and negatively associated with 1,000-m running time, indicating that students with better SLJ performance tended to have faster 1,000-m running performance. This finding suggests a relationship between explosive power and cardiorespiratory fitness performance in this sample. However, it should not be interpreted as evidence that explosive power improves cardiorespiratory fitness or that SLJ can predict aerobic capacity (Pérez-Ramírez et al., 2024). The association may be influenced by general physical activity level, training background, body size, or other unmeasured factors (Jing et al., 2025). Therefore, SLJ may be regarded as a fitness-related field test associated with endurance performance, rather than a substitute for direct cardiorespiratory fitness assessment.

Regarding BMI, SLJ performance was significantly and negatively associated with BMI-defined weight status. This result indicates that students with lower BMI values within the studied range tended to perform better in SLJ. However, BMI should not be interpreted as a direct measure of body composition or cardiometabolic risk in this study. Because BMI cannot distinguish fat mass from lean mass, a higher BMI may reflect greater adiposity, greater muscle mass, or both. In particular, greater lean mass may contribute positively to explosive performance, whereas excess non-functional mass may increase mechanical load during jumping. Therefore, the BMI-related findings should be framed strictly as associations between SLJ performance and weight status, rather than evidence of body composition differences or cardiometabolic health status (Melin et al., 2024; Ge et al., 2025).

From a public health and practical application perspective, the present findings suggest that SLJ may be useful as a standardized, low-cost, and easily administered field test associated with multiple physical fitness components (Song and Zhang, 2026). Its scalability and minimal equipment requirements make it potentially suitable for large-scale fitness assessment in school or university settings. However, SLJ should not be viewed as a comprehensive health assessment tool or a direct proxy for clinical health outcomes. Instead, it may serve as one accessible component within a broader physical fitness assessment system. In this sense, SLJ may contribute to more efficient and inclusive data collection, particularly when resources for comprehensive testing are limited.

Several limitations should be acknowledged. First, this study employed a cross-sectional design; therefore, causal relationships, predictive validity, and directionality cannot be established. Second, the sample was restricted to male college students, which limits the generalizability of the findings to females, other age groups, and non-college populations. Third, BMI was used only as an indicator of weight status and should not be considered a direct measure of body composition or cardiometabolic risk. Future studies should adopt longitudinal or intervention designs, include more diverse populations, and incorporate additional measures such as body fat percentage, lean mass, waist circumference, blood pressure, metabolic biomarkers, physical activity behavior, and mental health indicators. Such research would help clarify whether SLJ has broader value within scalable and equitable physical fitness assessment models.

## 5 Conclusion

Standing long jump (SLJ) performance was significantly associated with multiple health-related physical fitness components, including cardiorespiratory fitness, muscular endurance, flexibility, and body composition, highlighting its role as an integrated indicator of lifestyle-related physical fitness.

Within the framework of health data equity, SLJ demonstrates strong potential as a simple, low-cost, and scalable proxy for multidimensional health assessment. Its minimal equipment requirements and ease of implementation make it particularly suitable for large-scale population health monitoring, as well as for use in resource-limited settings where access to comprehensive assessment tools may be constrained.

By enabling efficient and standardized data collection, SLJ may contribute to reducing disparities in health assessment and support more inclusive and equitable health data generation. Its application in schools, communities, and public health systems provides a practical pathway for early identification of individuals at potential cardiometabolic and mental health risk.

Future research should adopt longitudinal designs, include more diverse populations, and incorporate multidimensional health indicators—including mental health and behavioral measures—to further validate and expand scalable and equitable health assessment models.

## Data Availability

The raw data supporting the conclusions of this article will be made available by the authors, without undue reservation.
